# Wernicke’s Encephalopathy in a Patient With Crohn’s Disease: A Case Report

**DOI:** 10.7759/cureus.45810

**Published:** 2023-09-23

**Authors:** Fatima Adamou, Doua Darkaoui, Hajar Koulali, Zahi Ismaili, Ghizlane Kharrasse

**Affiliations:** 1 Hepato-Gastroenterology, Mohammed VI University Hospital, Oujda, MAR; 2 Digestive Diseases Research Laboratory (DSRL), Faculty of Medicine and Pharmacy, Mohammed First University, Oujda, MAR; 3 Gastroenterology and Hepatology, Mohammed VI University Hospital Center/Mohammed First University, Oujda, MAR

**Keywords:** brain mri, triad, thiamine, crohn's disease, wernicke's encephalopathy

## Abstract

The inflammatory bowel diseases (IBD), including Crohn’s disease (CD) and ulcerative colitis (UC), are chronic inflammatory disorders of the intestine. Both are associated with intestinal and extra-intestinal manifestations (EIM). EIM are usually related to intestinal disease activity and may precede or develop concurrently with intestinal symptoms. Although they are well documented, the association of CD with neurological and neuromuscular involvement is rare and controversial, with sporadic and contradictory data regarding its prevalence and spectrum. Neurological involvement can affect the central or peripheral nervous system, with thrombotic events being the most frequent complication. Wernicke's encephalopathy (WE) is one of the neurological complications that occurs in the general population with a clinical prevalence ranging from 0.04% to 0.13%. Although no specific data exists for IBD patients, it is imperative for clinicians to be vigilant and consider the possibility of this condition even with mild neurological symptoms and to administer vitamin B1 promptly before attempting any biological assessment. Timely treatment is essential to avoid irreversible damage or even the death of the patient.

We report herein a challenging case of WE in CD and we discuss the literature.

## Introduction

Crohn’s disease (CD) is a chronic and debilitating inflammatory condition of the digestive tract [1] that impacts the quality of life. Its prevalence varies globally from one region to another, with an increasing incidence rate each year, making it a public health concern [2,3]. CD can lead to nutritional deficiencies resulting from reduced food intake, poor nutrient absorption, medication side effects, and systemic inflammation due to disease activity [4,5]. These deficiencies can cause severe neurological deficits, such as Wernicke's encephalopathy (WE), an under-recognized complication of inflammatory bowel diseases (IBD). Here we report a challenging case of WE in a patient followed in our gastroenterology department for CD.

## Case presentation

**Figure 1:**
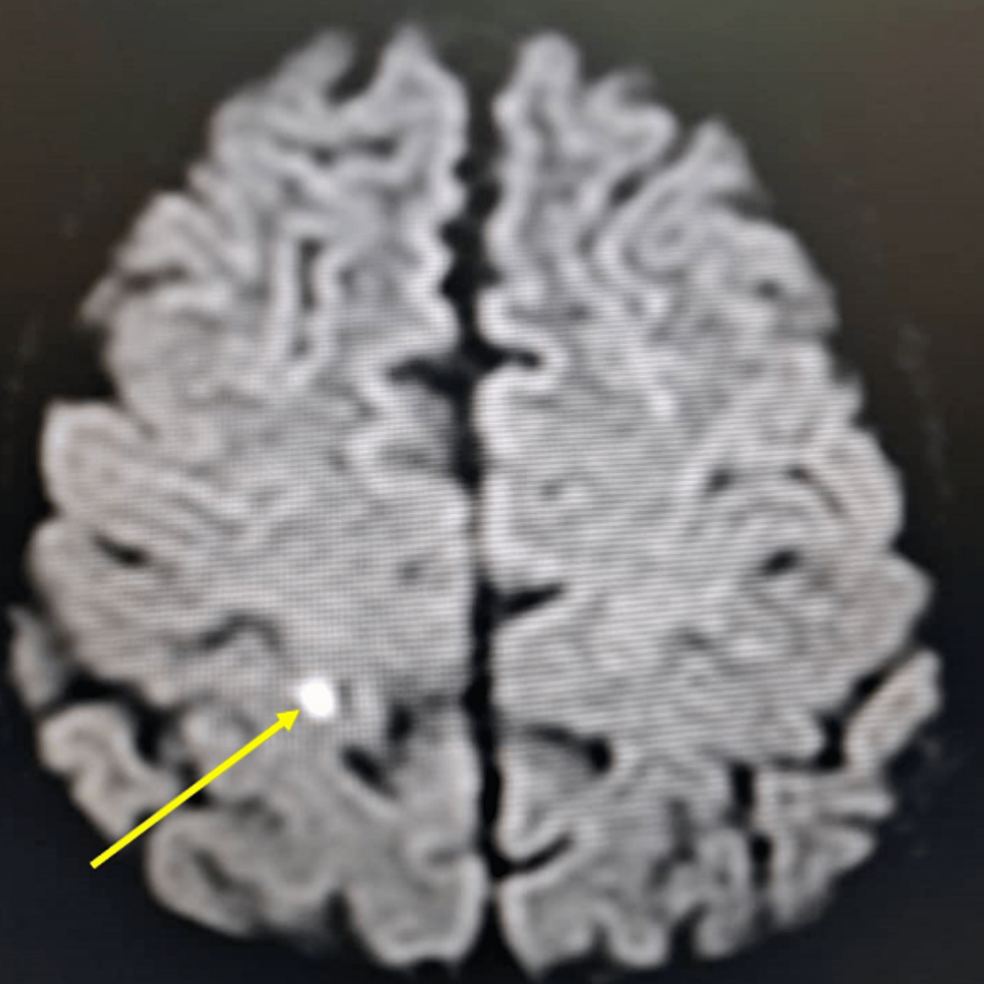
Brain MRI diffusion sequence showing small punctate signal abnormalities (hyperintensities) in the frontal and parietal lobes suggesting a lacunar ischemic stroke (embolic origin) but a WE is not to be ruled out. MRI: magnetic resonance imaging, WE: Wernicke's encephalopathy

This is a 38-year-old man, non-smoker, non-alcoholic, with a history of head injury in childhood, followed for 10 years for ileal stenosis CD, self-medicating with mesalazine at a dose of 4 grams/day. He underwent surgery for peritonitis (the cause of the peritonitis was not identified, it was a white laparotomy. There was pus in the surgical examination without knowing the origin) a month prior to his admission to our department. He was put on ceftriaxone at a dose of 3 grams/day but did not take metronidazole. The patient had an excessive use of steroids due to self-medication, leading to suspected adrenal insufficiency. The patient was admitted to the hospital for extremely sudden, periumbilical abdominal pain relieved by the passing of gas, along with a cessation of bowel movements four days prior to admission. Additionally, on the same day as the emergency room admission, the patient experienced generalized tonic-clonic seizures with spatial-temporal disorientation and confusion. Heart rate and blood pressure were normal, otherwise, the physical exam showed bilateral lower limb edema (extending to the thighs), body mass index (BMI) at 18 kg/m^2^. Laboratory tests showed hypoglycemia (low blood glucose) at 0.5 g/L, a potassium level was low at 2.1 g/l, normocytic normochromic anemia with hemoglobin levels at 9 g/dL, and mean corpuscular volume (MCV) at 90fl. The patient exhibited vitamin deficiencies, particularly vitamin D (<3.5 ng/L), vitamin B9 (3 ug/L), and vitamin B12 (160 pg/L), along with hyperferritinemia at 662 ug/L. Due to the patient's irregular steroid use and frequent episodes of hypoglycemia which was corrected with intravenous (IV) dextrose followed by infusion of glucose, a morning cortisol level test was performed, revealing a low level of cortisol: 32 ng/mL (reference range: 100-250 ng/mL), confirming adrenal insufficiency. He was placed on hydrocortisone 20 mg/day orally. The patient's neurological symptoms in particular the convulsive seizure and temporal spatial disorientation as well as the confusion and the mode of installation of these symptoms which were acute prompted a thorough examination to consider various possible causes, including hypoglycemia, as well as the potential of an ischemic stroke secondary to cerebral thrombosis or WE. The cranial MRI showed punctate signal abnormalities in the subcortical frontal and parietal white matter with diffusion hyperintensity values which suggest a lacunar ischemic stroke whose origin is embolic (pulmonary embolism) but a WE is not to be ruled out. The MRI found also a left porencephalic cavity (related to his previous head injury) and described a well-defined, regular, left parietal subgaleal hematoma showing fluid-like hyperintensity on T2-weighted images (Figure 1).

Considering the patient's malnourished state and concurrent active disease and the persistence of neurological signs even after the hypoglycemia correction, there was a strong suspicion of WE. To address this, the patient received Vitamin B1 at a dosage of 500mg three times daily for five days, followed by 500mg/day for another five days intravenously. Remarkably, within the first three days of treatment, the patient's consciousness gradually improved, confirming the diagnosis of WE.

**Figure 2:**
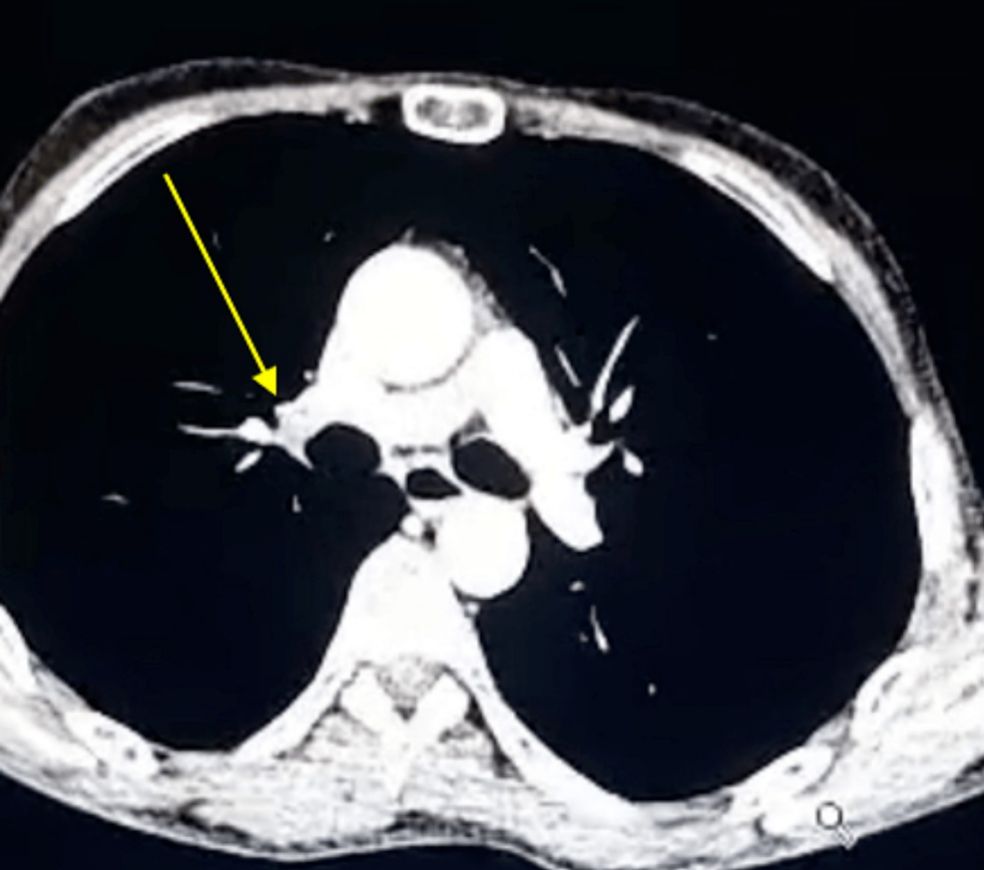
Axial thoracic CT scan (mediastinal window) showing a hypodense material in the right basal segment, consistent with a thrombus. CT: computed tomography

**Figure 3:**
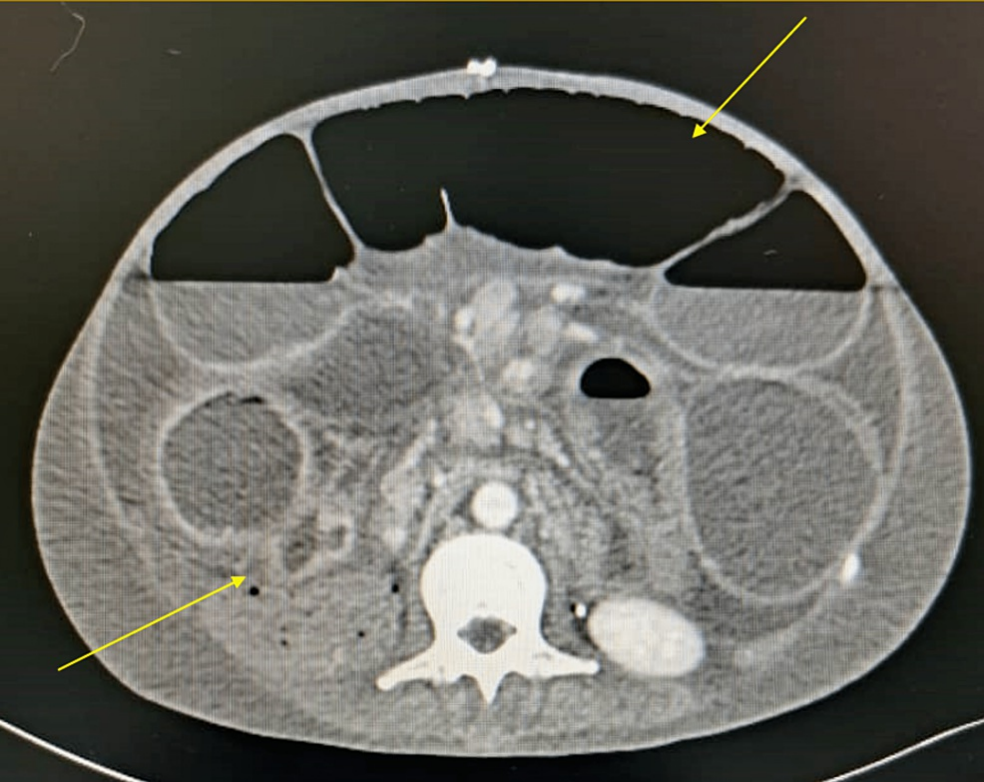
Abdominal CT scan with axial section showing a clear radiological occlusion with stenosis at 15 cm from the last ileal loop. CT: computed tomography

A thoracic and abdominal CT scan revealing the presence of a segmental basal right pulmonary embolism (Figure 2) associated with massive distension of the small bowel loops measuring 7 cm upstream of thickened walls of a stenotic ileal loop, arranged in layers (complete occlusion on imaging). There is also evidence of a stenotic image approximately 15 cm from the ileocecal valve (Figure 3). A laboratory test of vitamin B1 before the patient was given vitamin B1 found a low concentration at 10 nmol/l (low level). The patient had a CRP level of 70 mg/l and stool calprotectine at 600 ug/mg.

**Figure 4:**
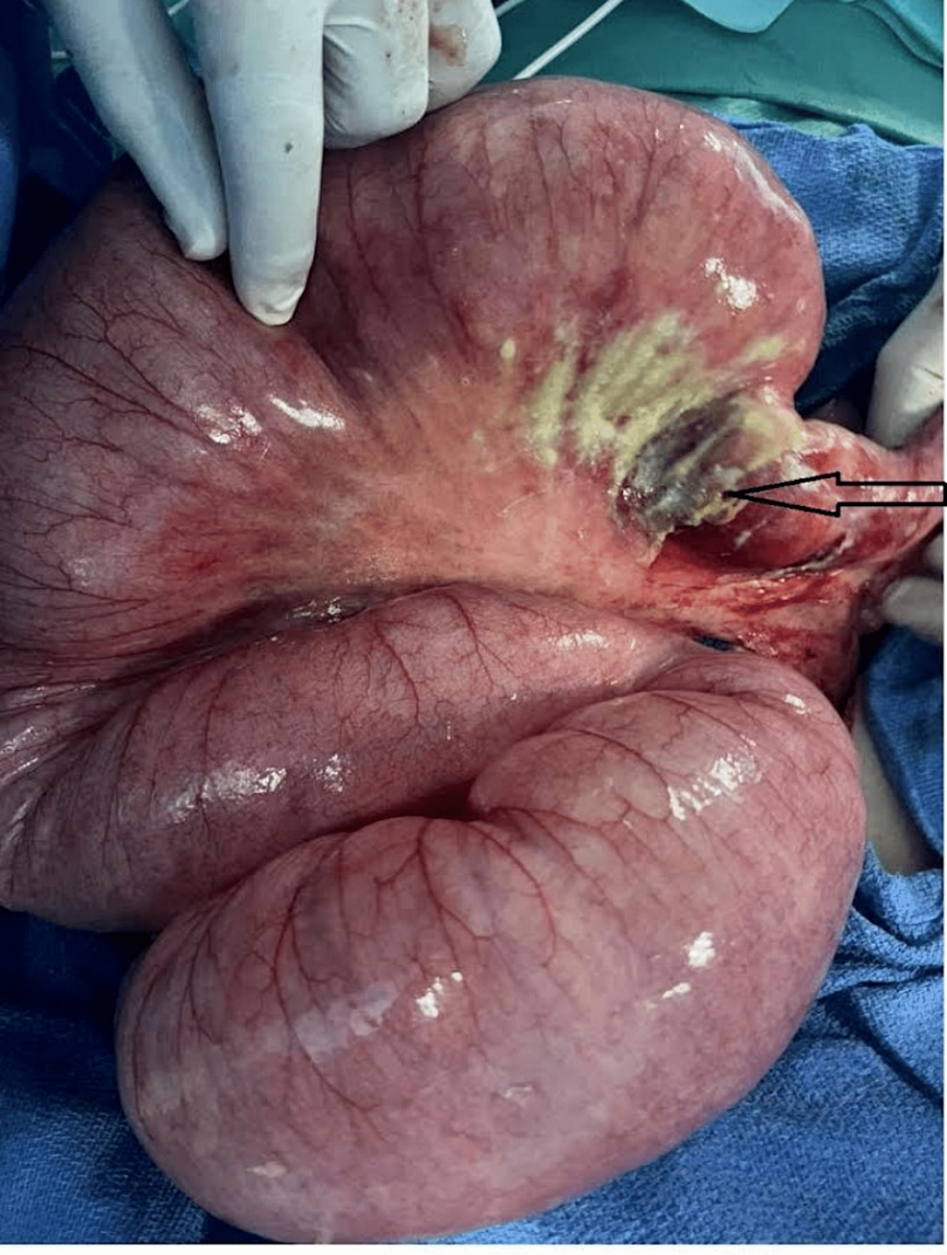
Intraoperative image showing a jejunal defect at 10 cm from the last ileal loop.

The patient was put on a high-calorie, high-protein, oral diet (Modulen® IBD (Nestle, UK) + low-residue diet) and was put on curative dose anticoagulation for his pulmonary embolism. IV steroids were not obtained, he was on hydrocortisone because he had adrenal insufficiency confirmed on morning cortisol levels. Daily physiotherapy sessions for the limbs were also provided and the patient underwent an ileocecal resection following a multi-disciplinary consultation meeting (Figure 4). He has shown improvement clinically and currently, he is undergoing adalimumab therapy as part of the treatment plan.​​​​​

## Discussion

CD is the most common type of IBD, with an incidence ranging from 0.97 to 57.9 per 100,000 in Europe, 8.8 to 23.14 per 100,000 in North America, and 0.15 to 6.5 per 100,000 in Asia and the Middle East [6-7]. In addition to affecting the gastrointestinal (GI) tract, IBD has several extra-intestinal manifestations (EIMs) such as uveitis, sacroiliitis, ... , accounting for approximately 25-40% of cases [8]. These EIMs can potentially affect multiple organ systems, including the skin, eyes, joints, bones, blood, kidneys, liver, and biliary tract [6]. Neurological involvement is seen in a range of 0.25% to 35.7%, although limited studies have been conducted on this aspect [4,9]. Among these neurological manifestations in IBD, WE is a rare condition, with a prevalence varying from 0.04% to 0.13%, contrasting with 0.8% to 2.8% based on autopsy studies, it’s still a rare and severe condition resulting essentially from an acute deficiency of thiamine [10]. The main cause of vitamin B1 deficiency is chronic alcoholism, accounting for 50% of cases. In our case, the patient didn't have a history of alcoholism, his vitamin B1 deficiency could be linked to his disease which was active. Neurologic deficits are common but often overlooked in IBD patients, either due to malabsorption mechanisms, surgery [11], or hydroelectrolytic losses (vomiting, diarrhea, leading to other hydroelectrolytic disturbances in these patients) [12]. This one could have caused vitamin B1 deficiency in our patient.

Other mechanisms may be involved in the development of general neurological symptoms in CD such as:

➢ Hypercoagulability related to chronic inflammatory response, which can lead to ischemic events, such as ischemic strokes, as seen in our patient, formation of toxic metabolic agents [4,5] in the damaged intestine.

➢ Immunological disturbances that may lead to an autoimmune reaction against glioneuronal components and opportunistic infections resulting from weakened immune systems or IBD treatments.

➢ Malabsorption and secondary deficiencies in vitamins (especially vitamin B1), essential for maintaining and regenerating myelin [4]. These mechanisms can cause neurological damage individually or in combination. Unfortunately, in many cases, identifying the primary pathological factor is challenging. Additionally, the pharmaceutical agents (Table 1) commonly used to treat IBD may also contribute to the development of encephalopathy.

**Table 1:** Summary of medications used in the treatment of IBD and potentially responsible for Wernicke encephalopathy. IBD: inflammatory bowel disease, WE: Wernicke's encephalopathy

Drug Families	WE Frequency
Antibiotics (metronidazole and ciprofloxacin)	Encephalopathy (in 1% of cases)
Cyclosporine	Encephalopathy (in 1/1000 cases)

WE is characterized by a classic triad: ophthalmoplegia, cerebellar ataxia, and confusion [4]. Untreated, WE can lead to severe neurological disorders, such as Korsakoff psychosis, and even death. However, It is essential to note that the classical triad of symptoms is only present in 16 to 33% of patients during the initial clinical examination and is complete in only 8 to 30% of cases [4]. Diagnosing this neurological condition can be challenging, delayed, or even only confirmed after the patient's death. Although the presence of oculomotor disturbances is highly suggestive, they are observed in only 15 to 29% of cases [6]. The diagnosis of WE primarily relies on clinical evaluation. In this regard, a comprehensive clinical approach is the best method to achieve an accurate diagnosis, and clinicians should consider WE in cases where a patient presents with unbalanced nutrition or subacute or chronic diseases associated with increased metabolism or changes in food intake or absorption, even if only one symptom of the classic triad is present, in our patient neurologic signs were not specific but the clinical context was suggestive, especially after the failure of the initial therapeutics.

When WE is suspected, brain MRI is considered a valid and preferable method for confirming the diagnosis [4,13]. It is more sensitive (53%) than CT scans and has a specificity of up to 93%. Typical manifestations include hyperintensities on T2, fluid-attenuated inversion recovery (FLAIR), and sometimes diffusion sequences. These anomalies are symmetric, and appear in the postero-medial thalamic nuclei on both sides of the third ventricle, the mamillary bodies, and the periaqueductal region [14-16]. Atypical locations may also occur, such as the head of the caudate nucleus, lentiform nuclei, and cortex, but these are always associated with classic anomalies. Diffusion sequences may reveal hyperintensity related to vagogenic or cytotoxic edema. Brain MRI is also useful in ruling out other diagnoses as the clinical presentation is not specific [16]. A normal MRI does not rule out the diagnosis of WE. Confirming the diagnosis requires measuring the thiamine concentration or assessing transketolase activity in red blood cells. However, these tests have low specificity and are not always available [17].

The cornerstone of WE treatment is the administration of thiamine as soon as the disease is suspected and as early as possible to prevent irreversible deficits, including Korsakoff syndrome and death. Clinicians should not wait for additional results before starting treatment because the confirmation of diagnosis is often difficult and delayed. According to the European Federation of Neurological Societies (EFNS) guidelines, the recommended thiamine dosage is 500 mg IV three times a day for three to five days, followed, if there is improvement after initial treatment, by 250 mg intravenously daily for a minimum of three to five additional days. Early administration of thiamine can improve symptoms, especially when done promptly. Neurocognitive symptoms such as apathy, drowsiness, and confusion respond well to treatment confirming the diagnosis. Furthermore, a delay or lack of recovery should alert physicians to consider other diagnoses [4,18].

## Conclusions

WE in patients with IBD is very rare and not well known. However, it is essential to note that in individuals with active IBD presenting with vomiting and/or diarrhea, thiamine supplementation should be necessary. In these situations, at the onset of the first symptoms of WE, prompt treatment with high doses of thiamine can save lives.
